# Atlas-based analysis of 4D flow CMR: Automated vessel segmentation and flow quantification

**DOI:** 10.1186/s12968-015-0190-5

**Published:** 2015-10-05

**Authors:** Mariana Bustamante, Sven Petersson, Jonatan Eriksson, Urban Alehagen, Petter Dyverfeldt, Carl-Johan Carlhäll, Tino Ebbers

**Affiliations:** Division of Cardiovascular Medicine, Department of Medical and Health Sciences, Linköping University, Linköping, Sweden; Center for Medical Image Science and Visualization (CMIV), Linköping University, Linköping, Sweden; Department of Clinical Physiology, Department of Medical and Health Sciences, Linköping University, Linköping, Sweden; Division of Media and Information Technology, Department of Science and Technology/Swedish e-Science Research Center (SeRC), Linköping University, Linköping, Sweden; Department of Cardiology, Department of Medical and Health Sciences, Linköping University, Linköping, Sweden

**Keywords:** 4D flow cardiovascular magnetic resonance (4D flow CMR), Flow volume, Image segmentation, Image registration, Phase contrast

## Abstract

**Background:**

Flow volume quantification in the great thoracic vessels is used in the assessment of several cardiovascular diseases. Clinically, it is often based on semi-automatic segmentation of a vessel throughout the cardiac cycle in 2D cine phase-contrast Cardiovascular Magnetic Resonance (CMR) images. Three-dimensional (3D), time-resolved phase-contrast CMR with three-directional velocity encoding (4D flow CMR) permits assessment of net flow volumes and flow patterns retrospectively at any location in a time-resolved 3D volume. However, analysis of these datasets can be demanding. The aim of this study is to develop and evaluate a fully automatic method for segmentation and analysis of 4D flow CMR data of the great thoracic vessels.

**Methods:**

The proposed method utilizes atlas-based segmentation to segment the great thoracic vessels in systole, and registration between different time frames of the cardiac cycle in order to segment these vessels over time. Additionally, net flow volumes are calculated automatically at locations of interest. The method was applied on 4D flow CMR datasets obtained from 11 healthy volunteers and 10 patients with heart failure. Evaluation of the method was performed visually, and by comparison of net flow volumes in the ascending aorta obtained automatically (using the proposed method), and semi-automatically. Further evaluation was done by comparison of net flow volumes obtained automatically at different locations in the aorta, pulmonary artery, and caval veins.

**Results:**

Visual evaluation of the generated segmentations resulted in good outcomes for all the major vessels in all but one dataset. The comparison between automatically and semi-automatically obtained net flow volumes in the ascending aorta resulted in very high correlation (*r*^2^=0.926). Moreover, comparison of the net flow volumes obtained automatically in other vessel locations also produced high correlations where expected: pulmonary trunk vs. proximal ascending aorta (*r*^2^=0.955), pulmonary trunk vs. pulmonary branches (*r*^2^=0.808), and pulmonary trunk vs. caval veins (*r*^2^=0.906).

**Conclusions:**

The proposed method allows for automatic analysis of 4D flow CMR data, including vessel segmentation, assessment of flow volumes at locations of interest, and 4D flow visualization. This constitutes an important step towards facilitating the clinical utility of 4D flow CMR.

**Electronic supplementary material:**

The online version of this article (doi:10.1186/s12968-015-0190-5) contains supplementary material, which is available to authorized users.

## Background

Cardiovascular magnetic resonance (CMR) based flow volume quantification in the great thoracic vessels is used in the assessment of several cardiovascular diseases such as valvular regurgitation and shunts [1]. Clinically, flow volume quantification is often performed based on semi-automatic segmentation of a vessel throughout the cardiac cycle in a manually positioned 2D phase-contrast (PC) CMR plane [2]. Three-dimensional (3D), time-resolved phase-contrast CMR with three-directional velocity encoding (4D flow CMR) permits accurate assessment of forward and backward flow volumes retrospectively at any location in a 3D volume over the cardiac cycle [3–6]. Advantages of 4D flow CMR compared to 2D cine PC-CMR are easy scan prescription, retrospective placement of analysis planes, and better comprehension of the cardiovascular physiology [7, 8]. Using 4D flow CMR, clinically useful measures such as Qp/Qs can be obtained from a single acquisition, eliminating the risk of physiological drift between different acquisitions and avoiding the risk of additional measurements due to invalid placement of 2D PC-CMR planes. Assessment of Qp/Qs with 4D flow CMR has been validated by comparison of the obtained ratios between different imaging modalities and assessment of the ratio in healthy volunteers [9–11]. Flow volume measurements in the proximal ascending aorta have also been compared with those obtained in the inferior and superior caval veins [12].

One of the main drawbacks of 4D flow CMR is the cumbersome and time-consuming analysis of the complex datasets generated. Additionally, the contrast between the blood flow and the surrounding tissues is low, which further aggravates the issue of manual segmentation in these datasets. Fully automatic methods for analysis of 4D flow CMR would improve the clinical utility of this promising technique.

Automatizing the process of blood flow visualization and quantification in the great thoracic vessels comes with a number of challenges, including segmentation of these vessels over the cardiac cycle, placement of 2D planes or 3D volumes for flow volume and peak velocity quantification, and visualization of interesting blood flow patterns using vector fields, streamlines, or pathlines. Some attempts have been made to automate this process: Three dimensional segmentation of vessels is important for orientation and visualization, and several solutions have been proposed for vessel segmentation of 3D angiographic data [13, 14]. Proposed semi-automatic methods for vessel segmentation in 4D flow CMR have focused on segmenting the vessels in 3D using a phase-contrast magnetic resonance angiography (PC-MRA) or a temporal maximum intensity projection (T-MIP) as a starting point for the segmentation [15–17]. This approach facilitates orientation in the 4D flow CMR dataset, but only yields segmentation of the vessels during systole as motion of the vessels over the cardiac cycle is not taken into account. Semi-automatic methods for vessel segmentation over the cardiac cycle have been developed for flow volume analysis of 2D phase-contrast MRI, but these techniques can not be applied directly to 4D flow MRI, which has less image contrast due to a lack of inflow effects. Furthermore, analysis of 4D flow CMR requires extensive user interaction in order to locate analysis planes [16, 18].

Atlas-based segmentation is a segmentation technique that has been used in a variety of fields across medical imaging [19–22]. The method involves deforming an already labeled image in order to extrapolate the labels to another unsegmented image. It is especially useful when segmenting anatomical structures instead of tissue types, such as in the presented problem of vessel segmentation. Using this approach, an analysis created for one dataset could be applied to another. For a 4D flow CMR dataset, this could include localization and segmentation of vessels of interest, placement of analysis planes or volumes of interest, and localization of emitters for particle trace visualization.

The aim of this study is to develop and evaluate a fully automatic method, based on atlas-based segmentation, for four-dimensional segmentation and analysis of 4D flow CMR data of the great thoracic vessels.

## Methods

### Study population

The proposed method was evaluated in a group of 21 subjects composed of 11 healthy volunteers (10 females, 1 male) with no history of prior or current cardiovascular disease or cardiac medication, mean age 67.3±3.6 years, range 60–72; and 10 patients (2 females, 8 males) with heart failure of different etiologies (ischemic cardiomyopathy and idiopathic dilated cardiomyopathy), mean age 61.1±13.4 years, range 32–78.

The patients were enrolled from the Department of Cardiology, Linköping University, Sweden. Exclusion criteria for the patients were: significant ventricular arrhythmia, heart rate below 40 bpm or over 100 bpm, cardiovascular shunt, or more than mild to moderate valvular disorder. The research was performed in line with the declaration of Helsinki and was approved by the Linköping ethics board, reference number 2010/273-31. All subjects gave written informed consent.

#### CMR examinations

CMR examinations were performed on a clinical 3T Philips Ingenia scanner (Philips Healthcare, Best, the Netherlands). All subjects received a gadolinium contrast agent (Magnevist, Bayer Schering Pharma AG) prior to the acquisition for a late gadolinium enhancement (LGE) study.

4D flow CMR datasets were acquired during free-breathing, using a navigator gated gradient-echo pulse sequence with interleaved three-directional flow-encoding and retrospective vector cardiogram controlled cardiac gating. Scan parameters included: Candy cane view adjusted to cover both ventricles, velocity encoding (VENC) 120 cm/s, flip angle 10°, echo time 2.6 ms, repetition time 4.4 ms, parallel imaging (SENSE) speed up factor 3 (AP direction), k-space segmentation factor 3, acquired temporal resolution of 52.8 ms, spatial resolution 2.7 × 2.7 × 2.8 mm^3^, elliptical k-space acquisition, scan time: 7–8 min excluding and 10–15 min including the navigator efficiency at heart rate 60 bpm.

Two-dimensional cine through-plane phase-contrast-CMR velocity data were acquired in a slice perpendicular to the main flow direction in the ascending aorta just downstream from the aortic valve, above the coronary arteries. The velocity data were acquired in a breath hold (duration approximately 10–15 sec) and the acquisition was retrospectively gated to the ECG using the following settings: slice thickness 8 mm, field of view (FOV) 350 mm × 300 mm, sensitivity encoding (SENSE) factor 2, velocity encoding range (VENC) 200 cm/s, echo time (TE) 2.2 ms, repetition time (TR) 3.8 ms, flip angle 10°. Five lines of k-space were acquired per heartbeat, resulting in a temporal resolution of approximately 20 ms.

The 4D flow CMR data were corrected for concomitant gradient fields on the scanner. Offline processing corrected for phase wraps using a temporal phase unwrapping method and background phase errors using a weighted 2nd order polynomial fit to static tissue [23, 24]. The 2D velocity data were corrected for concomitant gradient fields and background phase errors on the scanner. Following these processing steps, all datasets were screened for large residual background velocity offset errors that would affect the evaluation.

### Atlas-based segmentation and flow analysis

**Fig. 1:**
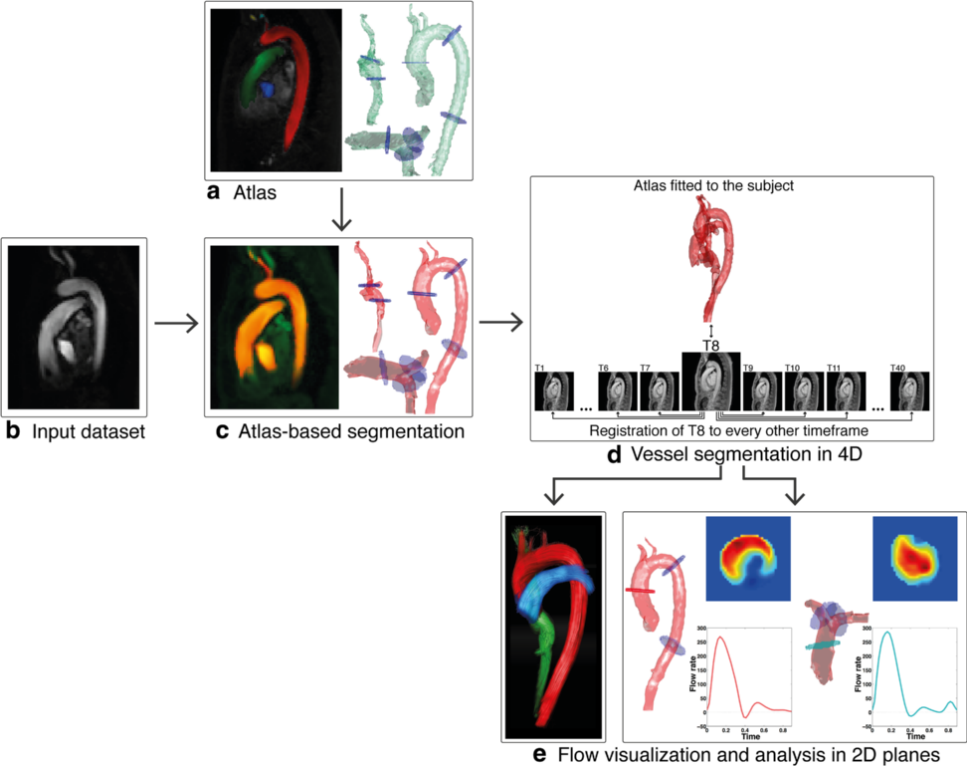
Overview of the proposed method. **a** An atlas is created from one healthy volunteer’s Phase Contrast Magnetic Resonance Angiography (PC-MRA), with 2D planes positioned at multiple locations of interest. **b** For each input dataset, a 3D PC-MRA is calculated. **c** The atlas is registered to the input dataset’s 3D PC-MRA in order to fit the vessels and planes from the atlas to the input dataset in one time frame in systole. **d** The magnitude time frame with the highest contrast in the PC-MRA is registered to every other time frame, the resulting deformations are then applied to the 3D segmentation in order to obtain a 4D segmentation of the vessels. **e** The flow in the segmented vessels can be visualized and automatically analyzed in each 2D plane

The proposed method utilizes atlas-based segmentation and is schematically illustrated in Fig. 1. Atlas-based segmentation enables automatic segmentation of specific regions-of-interest by registering an image volume in which these regions have been delineated i.e. an *atlas*, to an unsegmented image volume (hereafter termed *input dataset*). The deformed atlas will constitute a segmentation mask in the input dataset.

The atlas was created from one dataset of a healthy volunteer. The aorta, pulmonary artery, and vena cava were segmented semi-automatically. Additionally, the atlas includes the location of 2D planes to be used for flow analysis. The remaining datasets where analyzed using this atlas.

The method can be summarized as follows:

1. *Atlas-based segmentation:* The atlas is fitted to the input dataset by means of registration.
2. *4D vessel segmentation:* Segmentation of the great thoracic vessels in 3D over the entire cardiac cycle.
3. *Flow volume assessment:* Extraction of 2D vessel planes and net flow volume calculation in each plane.

The method was implemented in MATLAB (The MathWorks, Inc., Natick, Massachusetts, United States), using a registration toolkit for MATLAB that relies on non-parametric methods for non-rigid registration [25]. The analyses were performed on an HP Z820 Workstation with an Intel Xeon 6 Core 2.5 GHz processor and 64 GB RAM. Each step of the approach is described in more detail below.

#### Atlas creation

**Fig. 2:**
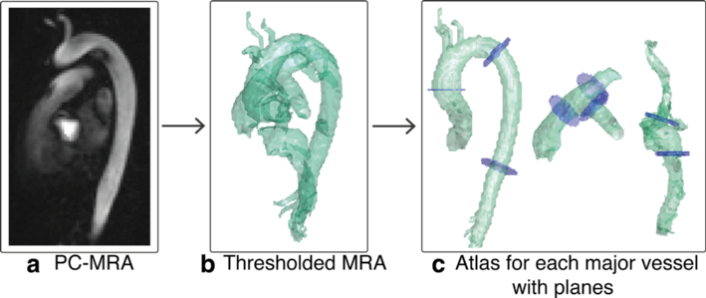
Atlas creation process. **a** A PC-MRA is created from the time frames in systole. **b** A rough segmentation of the major vessels is obtained by thresholding the PC-MRA. **c** The segmentation is adjusted manually for each vessel and 2D planes are added at specific locations

An atlas representing the great thoracic vessels was constructed by delineating each major vessel in one dataset belonging to a healthy subject. The segmentation needed to create the binary masks was performed in a systolic 3D phase-contrast Magnetic Resonance Angiography (PC-MRA) computed from 4D flow MRI data according to (1).

(1) $$  \text{PC\_MRA} = M \sqrt{{V_{x}^{2}} + {V_{y}^{2}} + {V_{z}^{2}}}  $$

where *V*_*x*_, *V*_*y*_ and *V*_*z*_ are the averages of the components of the three directional blood flow velocity over the systolic time frames, and *M* is the average magnitude of the signals acquired over the systolic time frames, which acts as a noise suppressor in areas of very low signal such as the lungs. The resulting PC-MRA is exemplified in Fig. 2a.

Additionally, 2D planes were defined at multiple locations of interest in the atlas, in this case: perpendicular to the ascending aorta, proximal descending aorta, distal descending aorta, pulmonary trunk, left and right branches of the pulmonary artery, inferior and superior vena cava. These planes were added in order to calculate the net flow volume passing through them over a heartbeat; and they also serve as a non-subjective, numerical way of evaluating the segmentation method.

The atlas creation process is demonstrated in Fig. 2. Note that the atlas only needs to be generated once, and it can be used subsequently to segment an arbitrary number of input datasets. Furthermore, the specific locations of the 2D planes described were selected for evaluation purposes; however, the method allows for any number of planes at any location close to the major vessels.

#### Atlas-based segmentation

For each input dataset, a 3D PC-MRA was calculated from 4D flow CMR data. The atlas’ PC-MRA was registered to each input dataset’s PC-MRA using affine registration followed by non-rigid registration. In this way, the robustness of the affine registration was used to obtain an initial rough deformation, while the accuracy of non-rigid registration was used for fine-tuning.

Both registration methods were performed using three scales, five iterations per scale, and linear interpolation. The non-rigid registration method used during this study was the Morphon algorithm, which uses local displacement estimations iteratively in order to fit a source image to a target image [26–28].

**Fig. 3:**
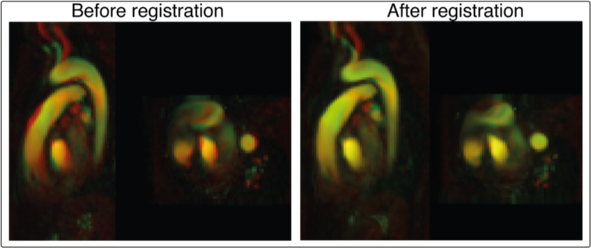
Registration of PC-MRAs. Sagittal and transverse planes of PC-MRA images of two different datasets before and after registration. One dataset is colored green and it is superimposed over the other in red

Diffeomorphic field accumulation was used in all registration instances, since it enables compression and deformation of the data, while avoiding tearing or folding. Both fluid and elastic regularization of the displacement field were also used in order to limit the amount of deformation permitted during the registration. Elastic regularization limits the deformation to model an elastic material, while fluid regularization models the deformation as a viscous fluid. A representative example of the appearance of the images before and after the registration process is shown in Fig. 3.

#### 4D vessel segmentation

After registering the atlas to the input 4D flow CMR dataset at systole, the atlas was adapted to all other time frames in order to account for the motion of cardiovascular structures over the cardiac cycle. This was achieved by applying non-rigid registration between the magnitude data of one specific systolic time frame and each of the remaining time frames. The systolic time frame chosen was the time frame with the highest signal magnitude, as this was the one that best resembled the fitted atlas obtained in the previous step. The resulting deformations were applied to the fitted atlas, generating a time-resolved segmentation that follows the motion of the vessels in the input dataset during the cardiac cycle.

**Fig. 4:**
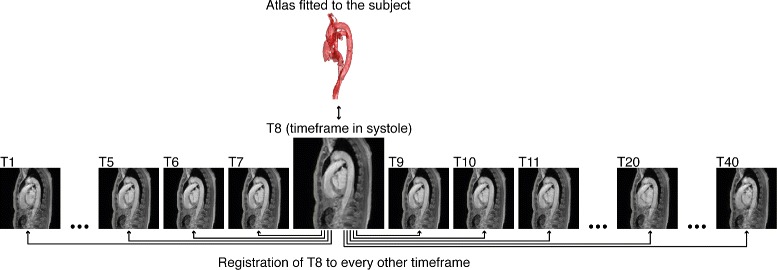
4D vessel segmentation. The systolic time frame of the magnitude image with the strongest signal amplitude in the PC-MRA (T8 in this case) is registered to each of the other time frames. The resulting deformations are then applied to the atlas, which has already been fitted to the subject

Non-rigid registration during this step was performed using five scales, five iterations per scale, and linear interpolation. The amount of scales was increased in order to make the registration more sensitive to smaller differences between the images. Both fluid and elastic regularization of the displacement field were used. Additionally, this registration method uses tensor magnitudes to calculate the edges of the shapes in the images and bases the process on these edges. While this approach is usually used when executing multimodal registration, our initial tests indicated that it was helpful in detecting the small differences between time frames in our data. The process described in this section is schematically presented in Fig. 4.

#### Flow volume quantification

The deformation obtained when fitting the atlas to the input dataset was also used to locate the 2D planes positioned in the major vessels. As the planes were deformed by this process, a flat plane was fitted into the points of each deformed plane using principal component analysis (PCA) [29]. The principal components resulting from the PCA were used as the vectors that define the location of the plane.

The fitted atlas was further improved in each vessel plane by using a 2D circular averaging filter with a nine pixel radius, thus making the vessel region rounder, smoother, and also a bit larger. Having a segmentation that includes a region slightly larger than the vessel doesn’t considerably affect the flow volume obtained since this value is not dependent on the vessel area, and the pixels that directly surround the vessel usually have very low velocities. This strategy guaranteed that none of the pixels with higher velocities in the vessel region were left out of the calculation.

**Fig. 5:**
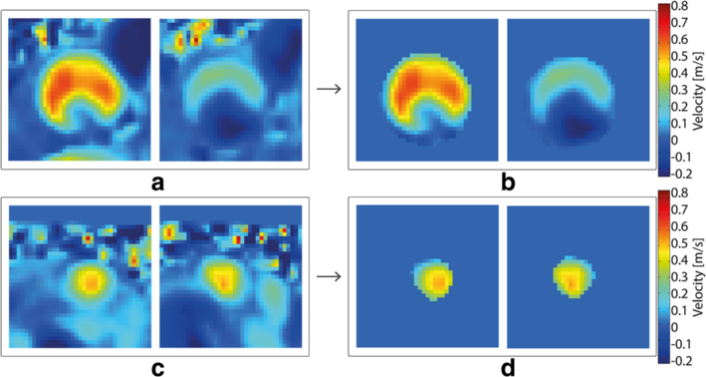
Vessel segmentation in 2D planes. Velocities [ *m*/*s*] through planes in the ascending aorta (**a**, **b**), and superior vena cava (**c**, **d**) in two different time frames during the cardiac cycle. **a** and (**c**) contain the original velocity information on the planes, while (**b**) and (**d**) are masked to account for vessel location and shape using the proposed method

The velocity data were extracted from each of the 2D vessel planes using linear interpolation in the 4D flow CMR data masked by the time-resolved atlas created in the previous steps. An example of the appearance of the mask in 2D and how it follows the vessels during the heartbeat can be seen in Fig. 5. Finally, the flow volume in each vessel plane was calculated by integrating the volumetric flow rates over the cardiac cycle.

### Evaluation

**Fig. 6:**
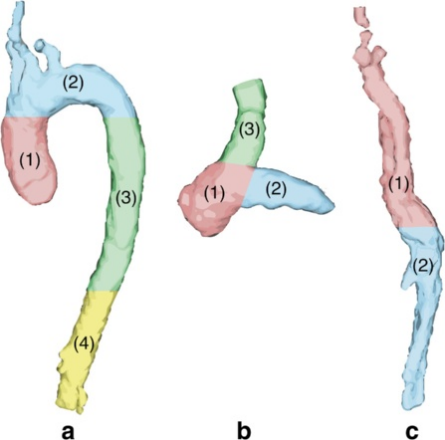
Vessel sections for visual evaluation. **a** Aorta divided into four sections: (1) ascending aorta, (2) aortic arch, (3) proximal descending aorta, (4) distal descending aorta; (**b**) pulmonary artery divided into three sections: (1) pulmonary trunk, (2) left branch, (3) right branch; and (**c**) caval veins divided into two sections: (1) superior vena cava, (2) inferior vena cava. Each section is shown in a different color

The accuracy of the results was evaluated visually and quantitatively for every input dataset. For the visual evaluation, each major vessel was divided into sections which were assessed and scored independently, as shown on Fig. 6. A numerical value between one and four was given as the score according to the following scale: 1 = poor segmentation, large errors are clearly visible; 2 = fair segmentation, intermediate errors visible; 3 = good segmentation, small errors visible; 4 = very good segmentation, no visible errors.

In the datasets where the segmentation was successful, net flow volumes obtained in the proximal ascending aorta with the proposed method were compared to those obtained in semi-automatically placed and segmented 2D planes. In order to determine the effect of the automatic positioning of the planes, the semi-automatically placed 2D planes were also segmented using the proposed automatic approach.

Additionally, flow volumes were calculated automatically at the following locations:

- Ascending aorta.
- Pulmonary trunk.
- Right and left pulmonary artery branches.
- Superior and inferior caval veins.

The net flow volumes at these locations are supposed to be closely related in subjects without cardiovascular shunts, which was also used to evaluate the proposed approach.

Furthermore, flow volumes in the proximal descending aorta and distal descending aorta were also calculated and compared between each other. The net flow volume is expected to only differ slightly between these two locations, as there are only minor branches in this section of a normal aorta.

Continuous variables are presented as mean ± standard deviation (SD). Simple linear regression analysis was used in order to evaluate the accuracy of the automatically calculated flow volumes versus the semi-automatically calculated ones in the ascending aorta. The same method was used to qualify the relationship between the flow volumes obtained at different vessel locations that are expected to be closely related to each other. A *p*-value <0.05 was considered statistically significant.

## Results

### Visual evaluation

**Fig. 7:**
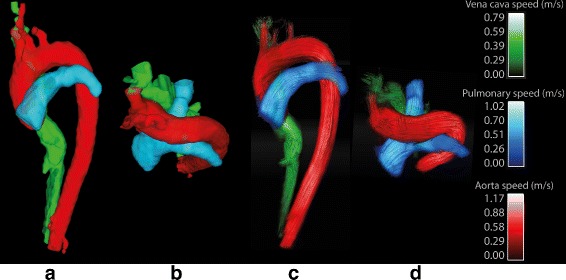
Resulting segmentation in systole. Near sagittal (**a**, **c**) and transverse (**b**, **d**) views of the segmentation of the great thoracic vessels. (**a**) and (**b**) show the resulting masks, while (**b**) and (**d**) show the speed (m/s) in each vessel at one time frame in systole. Each vessel is represented by a different color. A four-dimensional segmentation of the vessels can be viewed in the additional files provided by the authors [See Additional files 1 and 2]

An example of the resulting segmentation in a patient, including all the major vessels at one time frame in systole, together with streamline visualization of the blood flow is shown in Fig. 7, additional movie files show the segmentation and flow visualization in 4D [see Additional files 1 and 2]. Visual evaluation scores for all datasets can be seen in Table 1.

**Table 1:** Number of input datasets that received each score during visual evaluation of the segmentation obtained with the proposed method. Each vessel was divided into sections that were scored independently

Vessel section	Score = 1	Score = 2	Score = 3	Score = 4
Ascending aorta	1	1	1	17
Aortic arch	1	0	1	18
Proximal descending aorta	1	0	0	19
Distal descending aorta	3	0	0	17
Pulm. artery trunk	1	0	1	18
Pulm. artery left branch	1	0	1	18
Pulm. artery right branch	1	0	1	18
Superior vena cava	1	0	3	16
Inferior vena cava	1	4	1	14

Scale: 1 = poor segmentation, 2 = fair segmentation, 3 = good segmentation, 4 = very good segmentation. See Fig. 6 for a description of the selected vessel sections

Vessel segmentation was successful in 19 out of 20 input datasets analyzed, there were 19 segmentations as one of the 20 available datasets was used as the atlas. In one dataset, the subject’s cardiovascular morphology differed significantly from the morphology of the subject used as the atlas, thus producing an unreliable registration result. This dataset was not included in the net flow volume quantification evaluation.

### Evaluation through flow volume calculation

**Fig. 8:**
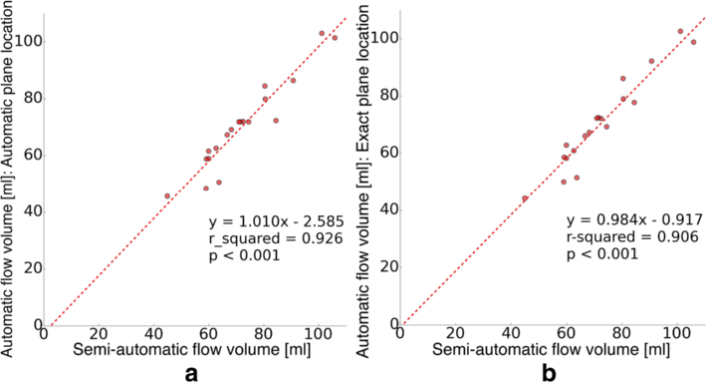
Semi-automatic vs. automatic vessel segmentation. **a** Linear regression analysis of flow volumes in the proximal ascending aorta obtained semi-automatically and automatically: *r* ^2^=0.926, *s* *l* *o* *p* *e*=1.01, *p*<0.001. **b** Linear regression analysis of flow volumes in the proximal ascending aorta obtained semi-automatically and with automatic segmentation on the exact same plane used during semi-automatic segmentation: *r* ^2^=0.906, *s* *l* *o* *p* *e*=0.984, *p*<0.001

A very strong relationship was found between auto- matic and semi-automatic flow volume calculation applied in the proximal ascending aorta: Semi-automatically versus automatically calculated net flow volumes (*r*^2^=0.926,*slope*=1.01,*p*<0.001, Fig. 8a), and semi-automatically calculated net flow volumes versus those calculated using automatic segmentation on the same plane used during semi-automatic segmentation (*r*^2^=0.906,*slope*=0.984,*p*<0.001, Fig. 8b). Mean ± SD of the net flow volumes obtained can be seen in Table 2.

**Table 2:** Net flow volumes obtained in the ascending aorta using 3 different approaches: semi-automatic, automatic, and automatic segmentation on the same plane located during manual segmentation (mean ± standard deviation)

Approach	Net flow volume (ml)
Semi-automatic	72.5±14.9
Automatic	71.3±15.7
Automatic on exact semi-automatic plane	70.4±15.4

**Fig. 9:**
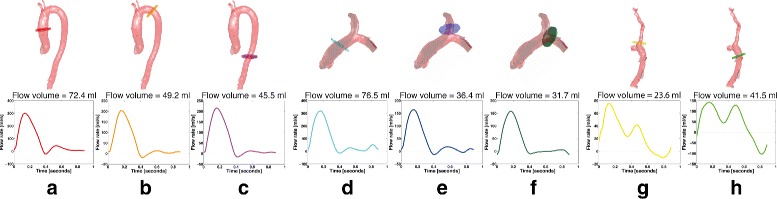
Flow analysis for one dataset. Illustration of flow analysis for one representative dataset. Net flow volume and volumetric flow rate perpendicular to the plane during the cardiac cycle are calculated for planes of interest in the (**a**) ascending aorta, (**b**) proximal descending aorta, (**c**) distal descending aorta, (**d**) pulmonary trunk, (**e**) right pulmonary branch, (**f**) left pulmonary branch, (**g**) superior vena cava, and (**h**) inferior vena cava

**Fig. 10:**
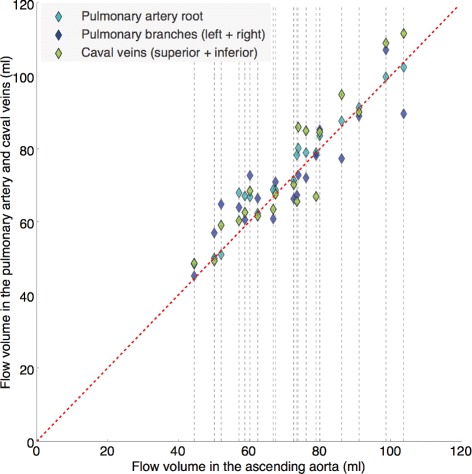
Flow volumes obtained automatically for each of the comparable values. Proximal ascending aorta versus pulmonary trunk (cyan), sum of the pulmonary branches (blue), and sum of the inferior and superior caval veins (green). Each column of values represents an input dataset and it is represented by a grey dashed line. The red dashed line shows the values of the flow volumes obtained in the proximal ascending aorta

Figure 9 shows an example of the flow analysis generated automatically for one input dataset, while Fig. 10 shows the flow volumes obtained automatically in the ascending aorta versus those obtained in the pulmonary artery and caval veins for all input datasets. It is worth noting that although these values should be closely related, they are not supposed to be exactly the same, even in a healthy cardiovascular system. Mean ± SD of the net flow volumes obtained at each location are shown in Table 3.

**Table 3:** Net flow volumes obtained in the great thoracic vessels (mean ± standard deviation)

Vessel	Net flow volume (ml)
Ascending aorta	71.3±15.7
Pulmonary trunk	73.9±14.9
Sum of pulmonary branches	72±13.5
Sum of inferior and superior caval veins	74±17.7

**Fig. 11:**
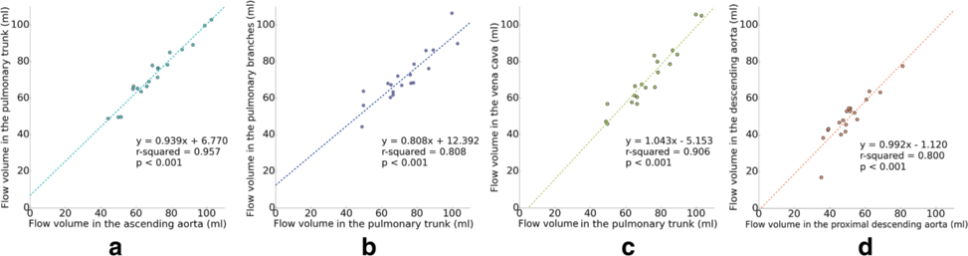
Linear regression analysis of flow volumes obtained automatically. **a** Proximal ascending aorta vs. pulmonary trunk (Qp/Qs): *r* ^2^=0.957, *s* *l* *o* *p* *e*=0.939, *p*<0.001. **b** Pulmonary trunk vs. pulmonary branches: *r* ^2^=0.808, *s* *l* *o* *p* *e*=0.808, *p*<0.001. **c** Pulmonary trunk vs. caval veins: *r* ^2^=0.906, *s* *l* *o* *p* *e*=1.043, *p*<0.001. **d** Proximal descending aorta vs. the descending aorta: *r* ^2^=0.8, *s* *l* *o* *p* *e*=0.992, *p*<0.001

Linear regression analysis was performed between the automatically measured flow volumes in the pulmonary trunk and proximal ascending aorta (Qp vs. Qs) resulting in a very strong relationship (*r*^2^=0.957, *slope*=0.939, *p*<0.001, Fig. 11a).

Flow volumes on the pulmonary trunk were also compared to the addition of the values obtained in the pulmonary branches (*r*^2^=0.808, *slope*=0.808, *p*<0.001, Fig. 11b), and the addition of the values obtained in the inferior and superior caval veins (*r*^2^=0.906, *slope*=1.043, *p*<0.001, Fig. 11c). These analyses resulted in strong and very strong relationships respectively.

Additionally, the regression analysis done between the proximal descending aorta and the descending aorta is shown in Fig. 11d, the result showed a strong relationship (*r*^2^=0.8, *slope*=0.992, *p*<0.001).

## Discussion

A method for automatic segmentation and flow assessment in the great thoracic vessels using 4D flow CMR data was developed and evaluated. The resulting segmentations were assessed visually, resulting in very good scores for all the great thoracic vessels. Comparison of net flow volumes from the proposed automatic method and the traditional semi-automatic segmentation of the vessels resulted in very strong relationships. Moreover, strong and very strong relationships were obtained between net flow volumes calculated automatically in vessels that are expected to be closely related to each other.

Several clinical protocols include assessment of flow volume from multiple locations of interest; complex congenital heart diseases such as total cavopulmonary connection, cardiovascular shunts, and regurgitation of semilunar valves. For such protocols, the total examination time can be reduced by utilizing 4D flow CMR with retrospective assessment of flow volumes at locations of interest in the 3D data volume [16, 18]. Traditionally, such approaches have required extensive user interaction during the segmentation process. The proposed automatic method simplifies the time-consuming analysis substantially, as no user interaction is needed in order to obtain a 4D segmentation and flow volume quantification at multiple locations.

The flow assessment in this study was focused on net flow volumes throughout the cardiac cycle. However, the proposed segmentation method could also be used to automatically assess a range of other measurements such as peak velocities, flow rates, pulse wave velocity, turbulent kinetic energy and pressure differences, all of which could be of value in research as well as in clinical practice.

When handling vessel motion, registration was done between the time frame with brightest PC-MRA and each of the other time frames. A possible alternative could have been to register each time frame to the next, since each time frame is probably more similar to the following one. However, errors in the registration of one time frame to the next would be accumulated during the entire process, making the final time frames less accurate, and also more strongly affected by the registration’s interpolation effects.

Initial execution time of the developed method for one dataset was around one hour, most of which was spent performing image registration; nevertheless, subsequent runs on the same subject usually take less than a minute since the registration results are saved during the first run. The specific version of the registration toolkit used in this study employs only CPU computing; however, it should be possible to obtain much faster results using GPU computing [30].

In the current study, an extracellular contrast agent was injected just prior to the acquisition in all of the patients and healthy volunteers, thus improving the contrast between the blood vessels and the background on the PC-MRAs created. The method was not extensively tested on 4D flow CMR images obtained without contrast agent, but preliminary results indicate that the approach can handle the expected lower contrast. Of note, many protocols in the clinical routine already include administration of contrast agents, e.g. contrast enhanced MRA imaging, perfusion, or delayed enhancement imaging.

The proposed approach was successful in all but one dataset. In this case, the initial registration of PC-MRAs was unsuccessful due to the fact that the dataset belonged to a patient with a severely dilated heart. Further improvements to the method may be necessary for application in patients with more severe cardiovascular abnormalities, such as complex congenital heart disease. In this study, one atlas was created, which was based on a dataset obtained from a healthy volunteer. Presumably, creating atlases also from subjects with different common pathologies could be useful in making the approach more robust. Moreover, the orientation of the planes with respect to the vessel direction could be improved by adding an extra step after the initial plane locations have been obtained, assuring the angle of the planes to be perpendicular to the vessel lumen. While minor offsets in plane angulations have minor impact on flow volume analysis, this exact plane angulations could be important when studying more advanced 4D flow CMR parameters.

Given the relatively low resolution of the 4D flow CMR datasets, the proposed segmentation method has only been evaluated in the great vessels of the cardiovascular system; and it is not expected to segment smaller vessels such as the brachiocephalic artery, the common carotid artery, or the left subclavian artery. The resolution of the datasets also hinders a very precise appraisal of the segmented areas, which motivated the evaluation through net flow volume assessment.

Possible future applications of the method could include analysis of a patient cohort with a broader spectrum of valvular disease severity and type. Moreover, evaluation of other parameters such as peak velocity in the lumen of specific vessel sections would also be most suitable in such a cohort.

## Conclusions

A method for automatic analysis of 4D flow CMR data of the great thoracic vessels data was developed and evaluated. In addition to 4D vessel segmentation, the method permits automatic assessment of flow volumes in any number of planes and 4D flow pattern visualization in the great thoracic vessels.

The proposed method is completely automatic once an atlas has been created. Therefore, it is not affected by the constraints of semi-automatic segmentation and can readily provide information about cardiovascular physiology and pathophysiology in a relatively short amount of time and more importantly, without any user interaction. This is a significant step towards achieving clinical utility of 4D flow CMR.

## Additional files

Additional file 1
**Vessel segmentation in 4D.** Resulting segmentation mask of the aorta (red), pulmonary artery (blue), and caval veins (green) over the cardiac cycle for one dataset. Visualization created with EnSight (CEI Inc.). (MP4 1126 kb)

Additional file 2
**Flow visualization in the segmented vessels.** Flow speed visualization in the aorta, pulmonary artery, and caval veins over the cardiac cycle using streamlines. Visualization created with EnSight (CEI Inc.). (MP4 1710 kb)

## Abbreviations

CMR: Cardiovascular Magnetic Resonance
PC: Phase-Contrast
MRA: Magnetic Resonance Angiography
T-MIP: Temporal Maximum Intensity Projection
VENC: Velocity Encoding
SENSE: Sensitivity Encoding
FOV: Field of View
CPU: Central Processing Unit
GPU: Graphics Processing Unit
